# The Baltimore College

**Published:** 1862-01

**Authors:** 


*The Baltimore College*, as might naturally be supposed, has suffered greatly by its proximity to the seat of war, and by its being cut off from that section of our country which was always its chief support. I have been informed, but do not assert it as a fact, that there is a class there of some *thirteen* students.

That the Cincinnati school should not be in active operation is greatly to be deplored, and the reflection upon the profession in the great West in allowing this school to languish is not to their honor or credit. It is to be hoped that when the troubles now existing in our country shall have been settled, this school will be revived and have an equal opportunity to perform its part in the education of those who are destined to fill up the ranks of the profession with competent practitioners.

